# The Impact of Interventional Weight Loss on Bone Marrow Adipose Tissue in People Living with Obesity and Its Connection to Bone Metabolism

**DOI:** 10.3390/nu15214601

**Published:** 2023-10-29

**Authors:** Michaela Tencerova, Gustavo Duque, Kerensa M. Beekman, Alessandro Corsi, Jeroen Geurts, Peter H. Bisschop, Julien Paccou

**Affiliations:** 1 Molecular Physiology of Bone, Institute of Physiology of the Czech Academy of Sciences, 14220 Prague, Czech Republic; michaela.tencerova@fgu.cas.cz; 2 Department of Medicine, Research Institute of the McGill University Health Centre, Montreal, QC H4A 3J1, Canada; gustavo.duque@mcgill.ca; 3 Department of Radiology and Nuclear Medicine, Amsterdam UMC, University of Amsterdam, 1105 AZ Amsterdam, The Netherlands; k.m.beekman@amsterdamumc.nl; 4 Department of Molecular Medicine, Sapienza University of Rome, 00161 Rome, Italy; alessandro.corsi@uniroma1.it; 5 Rheumatology, Department of Musculoskeletal Medicine, Lausanne University Hospital, 1011 Lausanne, Switzerland; jeroen.geurts@chuv.ch; 6 Department of Endocrinology, Amsterdam UMC, University of Amsterdam, 1105 AZ Amsterdam, The Netherlands; p.h.bisschop@amsterdamumc.nl; 7 Department of Rheumatology, MABLab ULR 4490, CHU Lille, University Lille, 59000 Lille, France

**Keywords:** bone marrow adipose tissue, obesity, weight loss, metabolic and bariatric surgery, osteoporosis, bone mineral density, imaging, fractures, clinical trials

## Abstract

This review focuses on providing physicians with insights into the complex relationship between bone marrow adipose tissue (BMAT) and bone health, in the context of weight loss through caloric restriction or metabolic and bariatric surgery (MBS), in people living with obesity (PwO). We summarize the complex relationship between BMAT and bone health, provide an overview of noninvasive imaging techniques to quantify human BMAT, and discuss clinical studies measuring BMAT in PwO before and after weight loss. The relationship between BMAT and bone is subject to variations based on factors such as age, sex, menopausal status, skeletal sites, nutritional status, and metabolic conditions. The Bone Marrow Adiposity Society (BMAS) recommends standardizing imaging protocols to increase comparability across studies and sites, they have identified both water–fat imaging (WFI) and spectroscopy (^1^H-MRS) as accepted standards for in vivo quantification of BMAT. Clinical studies measuring BMAT in PwO are limited and have shown contradictory results. However, BMAT tends to be higher in patients with the highest visceral adiposity, and inverse associations between BMAT and bone mineral density (BMD) have been consistently found in PwO. Furthermore, BMAT levels tend to decrease after caloric restriction-induced weight loss. Although weight loss was associated with overall fat loss, a reduction in BMAT did not always follow the changes in fat volume in other tissues. The effects of MBS on BMAT are not consistent among the studies, which is at least partly related to the differences in the study population, skeletal site, and duration of the follow-up. Overall, gastric bypass appears to decrease BMAT, particularly in patients with diabetes and postmenopausal women, whereas sleeve gastrectomy appears to increase BMAT. More research is necessary to evaluate changes in BMAT and its connection to bone metabolism, either in PwO or in cases of weight loss through caloric restriction or MBS, to better understand the role of BMAT in this context and determine the local or systemic factors involved.

## 1. Introduction

Treatment of obesity includes lifestyle modifications, metabolic and bariatric surgery (MBS), and medications [1]. Lifestyle changes, including dietary modifications combined with physical activity, should always be the first step in treating people living with obesity (PwO) [1]. MBS procedures, such as Roux-en-Y gastric bypass (RYGB) and sleeve gastrectomy (SG), should only be considered as a secondary intervention when lifestyle changes prove ineffective [2]. Until recently, the most consistently effective intervention for weight loss has been MBS [3,4]. Glucagon-like peptide-1 receptor agonists (GLP-1Ras), such as liraglutide and semaglutide, have recently been approved by the US Food and Drug Administration (FDA) and the European Medicines Agency (EMA) as medications for chronic weight management in PwO, offering hope for a revolution in obesity care [5,6].

Changes in bone metabolism following interventional weight loss in PwO have been the subject of numerous studies [7,8,9]. It is increasingly recognized that surgical weight loss has adverse effects on bone metabolism, leading to elevated levels of bone turnover markers (BTMs) and decreased bone mineral density (BMD), as measured by dual-energy X-ray absorptiometry (DXA) [7,8]. The extent of high turnover bone loss implies more significant bone impairment following MBS compared to non-surgical weight loss [7,8]. This is probably related, at least in part, to the magnitude of weight loss following MBS. Following RYGB, PwO also experience substantial deterioration in bone microarchitecture and strength [10]. Undoubtedly, surgical weight loss is linked to a higher fracture risk [11,12]. Accumulating evidence indicates that RYGB is associated with a more pronounced reduction in BMD, a greater increase in BTMs, and a higher risk of fragility fractures compared to SG [13]. Intervention studies provide evidence that weight reduction in PwO through caloric restriction alone, or in combination with low-impact aerobic exercise, leads to increased levels of BTMs and reduced BMD, as assessed by DXA [9,14]. As previously reported, weight loss through caloric restriction did not consistently alter bone metabolism, and the extent of weight loss may be associated with changes in BTMs and BMD [9,14]. 

While the underlying mechanisms behind bone alterations following interventional weight loss in PwO remain incompletely understood, multiple factors are involved, including nutritional factors, reduced mechanical loading, loss of muscle mass, age, and changes in the secretion of gut hormones (GLP-1, peptide YY (PYY), glucose-dependent insulinotropic polypeptide (GIP)) and adipokines (adiponectin, leptin) [8,13]. It has been postulated that changes in bone marrow adipose tissue (BMAT), following weight loss in PwO, could contribute to adverse bone effects [15,16]. Indeed, recent reviews have emphasized the role of BMAT as a regulator of skeletal homeostasis [17,18]. However, the impact of dietary modifications and MBS on BMAT remains unclear, and the factors associated with changes in BMAT, as well as their association with bone metabolism, remain undetermined [15,16,17,18]. 

This review is centered on providing physicians with insights into BMAT and bone health in the context of interventional weight loss in PwO. This review has been divided into four parts. The first section introduces this unique fat depot and discusses the methods employed to assess, non-invasively, BMAT in humans. The second section provides an update on BMAT assessment in PwO. The third part focuses on changes in BMAT following non-surgical weight loss, while the final section presents BMAT changes after MBS. Furthermore, this review incorporates animal data to bridge gaps in human research and offer mechanistic insights that may be relevant to human study results. Finally, anorexia nervosa, another disease associated with changes in BMAT, is beyond the scope of this review. 

## 2. Human BMAT Is a Unique Fat Depot

**Figure 1:**
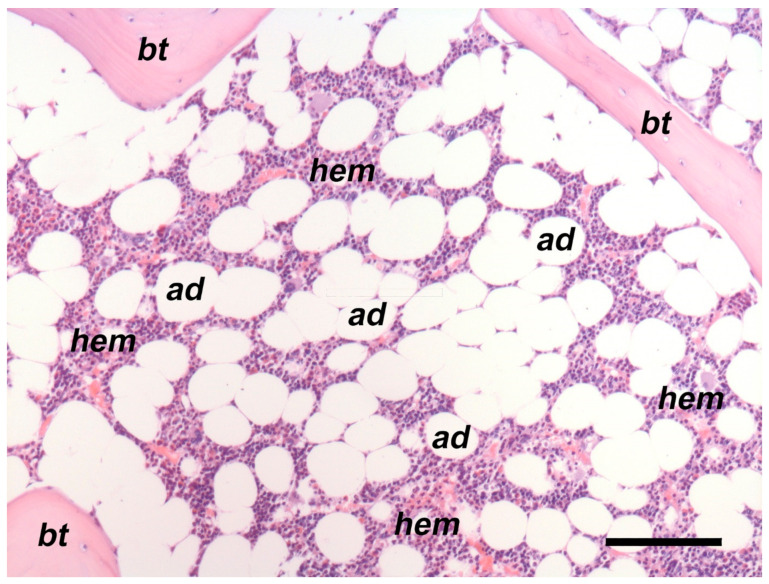
Representative histological image of the human bone/BM. As paraffin embedding determines dissolution of the lipids, BMAds appear as “ghosts” within the marrow. About 60% of the illustrated marrow is occupied by BMAds (ad). Formalin-fixed, decalcified, and paraffin-embedded adult BM biopsy. Haematoxylin–eosin-stained section. Bar: 100 µm; bt: bone trabecula; hem: hematopoietic cells.

BMAT is a specialized fat depot, distinct from other fat depots, residing within the bone marrow (BM). It physiologically expands during post-natal growth in a centripetal pattern, starting from the distal skeleton of hands and feet. Notably, there is growing recognition of the mutual influence between BMAT and bone [18,19]. BMAT originates from bone marrow stromal cells (BMSCs), which have the capacity to differentiate into various cell types, including bone marrow adipocytes (BMAds) and osteoblasts. In healthy adults, BMAT occupies 50–70% of the BM cavity (Figure 1) and constitutes approximately 10% of total body fat mass [20]. Aging and estrogen deficiency, related to the menopause, play pivotal roles in the pathophysiology of osteoporosis, and are linked to BMAT expansion in both long bones and vertebrae [18,21]. Clinical studies have indicated that higher amounts of BMAT may have adverse effects on bone health [22,23]. In fact, recent research suggests that BMAds may serve as an energy source for both the marrow and bone during stressful circumstances [24,25]. Recent animal studies demonstrated the presence of two types of BMAds: ‘regulated’ BMAds interspersed in proximal and axial parts of the skeleton, and ‘constitutive’ BMAds predominately present in the distal part of the skeleton. Regulated and constitutive BMAds show different metabolic activity in rodents [26]. However, their presence in humans still requires further investigation. Overall, the relationship between BMAT and bone is complex and subject to variation based on factors such as age, gender, menopausal status, skeletal sites, nutritional status, and metabolic conditions, including diabetes mellitus.

## 3. Non-Invasive Measurement of BMAT in Humans

Historically, the primary tool for evaluating bone and BM has been histology, with histomorphometry being a key clinical tool for analyzing both bone and BMAT. As imaging technologies such as magnetic resonance imaging (MRI) and computed tomography (CT) have advanced, it is now possible to directly and non-invasively study the role of BMAT in bone metabolism [27,28]. Both MRI techniques and dual-energy computed tomography (DECT) can be used to quantify the BM fat fraction (BMFF), which is a generic term for the estimate of the relative BM fat content [29]. MRI techniques can be used to quantify BMAT as the signal fat fraction (SFF), which is the ratio of the fat signal to the sum of the fat and water signal [29]. Both BMFF and SFF are dependent on scanning parameters and are, therefore, scanner and hospital dependent. When confounding factors, like T2* decay, T1 bias, and the multipeak fat spectrum, are considered when quantifying BMAT with MRI techniques, the proton density fat fraction (PDFF) can be calculated, which is the ratio of the unconfounded fat signal to the sum of the unconfounded fat and water signal [29]. Both MRI techniques like single/voxel proton magnetic resonance spectroscopy (^1^H-MRS) and water–fat imaging (WFI), or chemical shift encoding-based WFI (CSE-WFI), can be used to quantify the PDFF. Although ^1^H-MRS has traditionally been considered the gold standard, WFI is a robust alternative and has gained prominence [21,27]. Additionally, MRI can assess lipid saturation and unsaturation, providing insight into marrow adiposity composition, such as the presence and type of hydrogen bonding in the BM [27,28]. Compared to WFI, disadvantages of ^1^H-MRS are the small sampling area, the relatively long scanning times, and the expertise required to post-process the acquired spectra.

Accurate and standardized methods for quantifying BMAT are essential to evaluate its clinical implications. Understanding the differences between these methods is crucial for comparing results across different studies [29]. Despite significant research in BMAT imaging in humans, there remains a need to standardize imaging protocols and nomenclature, in order to increase comparability across studies and different skeletal sites. To address this, we recommend following the guidelines provided by the Bone Marrow Adipose Society (BMAS) [29]. A multicenter study on BMAT imaging in humans using both WFI and ^1^H-MRS is currently underway in France (NCT05005039). 

Due to space constraints in this manuscript, we refer readers to other reviews on the same topic, which cover other BMAT imaging modalities, including dual-energy CT (DECT), 18F FDG-PET, and HRpQCT [17,27,29]. Dual-energy CT (DECT) and HRpQCT can be used to quantify BMAT and BMD at the same time. However, exposure to ionizing radiation makes these methods less suitable for repeated quantification of BMAT, especially in younger subjects. 

## 4. Obesity and BMAT 

Animal models have been extensively used to investigate the relationship between BMAT and BMAds and bone homeostasis [20,24]. BMAT expands consistently in diet-induced obese models [30,31,32,33]. Interestingly, obesity is a condition associated with skeletal changes [7,34]. Due to the accumulation of lipids in all adipocytes through the different fat depots, it is not surprising that BMAT expands under a high-fat diet (HFD) regimen in rodent models [30,31,32,33]. Although BMAT expansion has been reported to occur consistently in HFD animal models, the effects on bone have been reported to be different [30,31,32,33] reflecting differences in the experimental conditions, including species, strain, and gender of the selected animal, type of diet, and starting age of HFD feeding [35,36]. Exercise has been reported to reduce BMAT expansion and improve bone quality in HFD-induced obesity. For humans, skeletal changes associated with obesity are more consistent. Obesity has long been thought to be protective against osteoporosis due to the fact that PwO present with normal and even higher BMD, as a result of mechanical adaptations to increased body weight [37]. However, several studies have challenged this assumption [7,38,39,40]. PwO have impairments in the quality of the bone matrix and structure, decreased levels of bone remodeling, and increased fracture risk at distinctive skeletal sites (proximal humerus, ankle, and upper leg). These latter effects on bone metabolism are largely attributed to chronic inflammation and hormonal disturbances linked to increased (central) adiposity [7,38,39,40]. BMAT may contribute to some of the deteriorating factors related to bone quality impairment in obesity, but there are not many clinical studies measuring BMAT in PwO and its association with bone metabolism [40,41,42,43]. 

Using histomorphometry in labeled transiliac bone biopsies, Cohen et al. evaluated bone microarchitecture, remodeling, and BMAT in healthy premenopausal women of various weights [40] (Table 1). They also measured BMD and trunk fat (a surrogate marker of visceral adipose tissue (VAT)), by DXA, in those 40 premenopausal women (37.3 ± 8.2 years), with a BMI ranging from 20.1 to 39.2 kg/m^2^. Compared to those in the lowest tertile of trunk fat (*n* = 13, BMI 21.6 ± 1.3 kg/m^2^), those in the highest tertile (*n* = 13, BMI 29.8 ± 4.6 kg/m^2^) had inferior bone quality (lower trabecular bone volume and higher cortical porosity) and stiffness, and markedly lower bone formation. Transiliac crest bone biopsies revealed that women in the highest tertile of trunk fat had similar BMAT levels compared to those in the lowest tertile: adipocyte number (#/mm^2^) 165.9 ± 39.8 versus 164.6 ± 35.9 (*p*-value adjusted for age = 0.30) and adipocyte volume/marrow volume (%) 27.9 ± 8.1 versus 22.6 ± 5.5 (*p*-value adjusted for age = 0.40). However, the authors concluded that bone marrow adiposity (BMA) (adipocyte volume/marrow volume) tended to be higher in the highest tertile, was significantly higher when the two upper tertiles were combined and correlated directly with trunk fat (r = 0.37, *p* = 0.019) in regression analyses. They also hypothesized a potential role for insulin-like growth factor 1 (IGF-I) as a mediator of the relationship between fat (trunk fat and BMA) and bone, as suggested previously by Bredella et al. [41].

Using ^1^H-MRS, Bredella et al. have compared BMAT at the lumbar spine in premenopausal women (*n* = 47, mean age of 32.8 ± 7.1 years) with high and low VAT (BMIs of 34.4 ± 4.9 versus 24.8 ± 5.1 kg/m^2^, respectively) [41]. Subjects with high VAT had significantly higher BMAT than those in the low VAT group (66.5% ± 26.0% versus 52.5% ± 21.2%; *p* = 0.05), and there was a mild positive correlation between BMAT and VAT (r = 0.34, *p* = 0.02), which remained significant after controlling for BMD (*p* = 0.05). Regarding bone quality, a negative correlation was found between BMAT and trabecular volumetric BMD (Tb.vBMD) at the lumbar spine using computed tomography (r = −0.39, *p* = 0.007), which remained significant after controlling for VAT (*p* = 0.03). There was also an inverse association between BMAT and IGF-1 (r = −0.39, *p* = 0.007). The authors found a significant difference in BMAT in premenopausal women with low and high VAT, whereas there was no difference in Tb.vBMD at the lumbar spine, suggesting a distinct role of BMAT as a mediator of bone impairment given the negative correlation between BMAT, Tb.vBMD, and IGF-1 [41].

Another study performed by Bredella et al. [42], comprised 106 healthy young men and women (mean age, 33.7 years ± 6.8; mean BMI, 33.1 kg/m^2^ ± 7.1) who underwent ^1^H-MRS of the L4 vertebrae. The main purpose was to investigate the associations between ectopic lipid levels, serum lipid levels, and BMAT. The bone parameters were not analyzed, which limits the interpretation of this study. Regardless of this limitation, L4 vertebral BMAT (lipid/water ratio) did not differ significantly (*p* = 0.07) between PwO (*n* = 88, 0.75 ± 0.38) and normal-weight controls (*n* = 18, 0.57 ± 0.20), but BMAT tended to be higher in PwO. 

Finally, in a study performed by Singhal et al. [43], 60 female adolescents and young adults between the ages of 14–21 years, 45 with obesity and 15 normal-weight controls, were evaluated. The authors had previously demonstrated inverse associations of BMAT with Tb.vBMD, both measured at the distal tibia in a small cohort of young adults and adolescents (mean age of 17.8 ± 2.1 years) with obesity (median BMI of 41.3 kg/m^2^) [44]. In this study, the assessment of BMAT was conducted at the L4 vertebrae and mid-femoral diaphysis using ^1^H-MRS. In contrast to previous findings, the lumbar and femoral BMAT (lipid/water ratio) was lower in adolescent girls with obesity compared to normal-weight controls: 0.39 ± 0.19 versus 0.59 ± 0.25 (*p* = 0.0039) at the lumbar spine, and 3.27 ± 2.41 versus 6.10 ± 1.69 (*p* < 0.0001) at the mid-femoral diaphysis. However, after controlling for weight, inverse associations of marrow adiposity with total tibial vBMD, and radial Tb.vBMD, were found.

While HFD leads to BMA expansion in preclinical models [30,31,32,33], the factors leading to decreases in BMA in adolescents and young adults with obesity are still unknown [43,44] and merit further research, as the opposite trend was observed in young men and premenopausal women [40,41,42]. 

Fazeli et al. [25] investigated BMA changes in 23 healthy volunteers (*n* = 10 women, mean age 33.3 ± 1.4 years, mean BMI 26.0 ± 0.3 kg/m^2^) in response to a structured, acute, short-term weight gain protocol, to achieve a 7% weight gain during the 10-day admission. Assessment of the lipid/water ratio was conducted at the L4 vertebrae, femoral metaphysis, and diaphysis, using ^1^H-MRS. The lipid/water ratio at the L4 vertebra, but not at the femoral metaphysis and diaphysis, increased significantly at 10 days (median (IQR) 6.7% (−1.7%, 40.2%), *p* = 0.02). They demonstrated that a high-calorie diet rapidly induced a marrow inflammatory immune response (increased in TNF-α expression in BMAds), like what is observed in other adipose depots, and confirmed that BMAT is dynamic and responsive to nutrient cues with region-specific variation. However, changes in BMAT and immunomodulatory factors observed during acute weight gain may not be similar and transposable to what happens during progressive weight gain and chronic obesity. 

**Figure 2:**
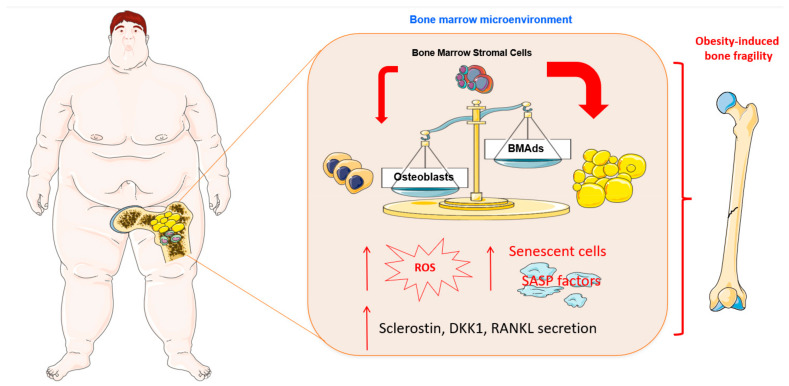
Obesity leads to changes in the composition of bone marrow cells affecting their differentiation potential and secretion of bioactive molecules. Obesogenic condition in bone marrow microenvironment causes expansion of bone marrow adipocytes (BMAds), leading to increased secretion of ROS and senescence-associated secretion phenotype (SASP) factors. In addition, obesity induces increased secretion of factors such as sclerostin, Dickkopf Wnt signaling pathway inhibitor 1(DKK1), and receptor activator of nuclear factor kappa-Β ligand (RANKL), impairing bone formation. All these obesity-induced changes in the bone marrow microenvironment contribute to increased bone fragility. Cell animations were adapted from SERVIER Medical Art; https://smart.servier.com, accessed on 20 October 2023, was used to create the figure.

In summary, clinical studies measuring BMAT in PwO are limited and have shown contradictory results. Comparing BMAT levels in PwO with normal-weight controls did not show consistent findings, which is at least partly related to the differences in the study population (premenopausal women, young men and women, and adolescent girls), the methods used to measure BMAT (^1^H-MRS and histomorphometry), and the measurement site (vertebrae and femoral diaphysis). However, BMA tended to be higher in patients with the highest VAT, and inverse associations between BMA and BMD have been consistently found. This suggests that local and systemic factors from BMA may impact bone health, and require further investigation (Figure 2).

## 5. Dietary-Induced Weight Loss and BMAT

With animal studies that clearly demonstrate higher levels of BMAT in HFD rodent models [30,31,32,33], it would be expected that dietary-induced weight loss interventions would also impact BMAT levels. Regarding the appropriate weight loss targets that have been associated with beneficial health outcomes, both the Centers for Disease Control and Prevention and the National Institute for Health and Care Excellence [45,46] recommend that an intervention should encourage individuals to set a weight loss target of 5–10% of their initial body weight. An additional target that demonstrates effectiveness is maintaining this body weight for at least one year [47]. 

Overall, the evidence demonstrating the effect of dietary-induced weight loss on BMAT is minimal [48,49,50,51]. This is primarily because most of the published trials are focused on the effect of this intervention on weight loss and other cardiometabolic variables, with very few groups looking at changes in BMAT as an outcome [48,49,50,51]. 

Cordes et al. [48] evaluated the changes in the BMAT in PwO after dietary intervention and compared them with changes in abdominal fat, liver fat, and serum lipids. Twenty women with obesity (mean age 47.0 ± 11.4 years and mean BMI 34.9 ± 3.8 kg/m^2^) participated in a 4-week dietary intervention of 800 kcal/d, plus additional vegetables. Abdominal 3T MRI was performed to measure changes in the subcutaneous adipose tissue (SAT) and VAT, and ^1^H-MRS was used to measure BMAT changes at the L5 vertebrae. After the 4-week dietary intervention, there was a statistically significant mean weight loss of 7.2 ± 1.6% and a decrease in BMI of 7.0 ± 1.8%. The greatest relative change after dietary intervention was found in the liver (−40.3% regarding fat content), followed by VAT volume (−15.1%), serum lipids (−12.6 to −14.5%), and SAT volume (−8.5%). There were no statistically significant changes in BMAT after the dietary intervention (43.1% to 42.5%, −1.1%, *p*  =  0.39). 

In another study by Vogt et al. [49], 29 patients with obesity and diabetes (10/19 men/women, median age: 59.0 years, median BMI: 34.0 kg/m^2^) prospectively joined a standardized 15-week weight loss program (six weeks of formula diet exclusively, followed by the reintroduction of regular food, with gradually increasing energy content over nine weeks). The MRI protocol included CSE-WFI before the program, at the end of the six weeks of formula diet, and at the end of the program at 15 weeks. The fat fractions of the abdominal organs, vertebral BMAT, and volumes of VAT and SAT, were determined. The median BMI decreased significantly from 34.0 kg/m^2^ to 29.9 kg/m^2^ (*p* < 0.001) at 15 weeks. The liver fat content was normalized (14.2% to 4.1%), and the vertebral PDFF decreased significantly throughout the program (57.5% to 53.6%, *p* = 0.018), while the fat content of the pancreas (9.0%), spleen (0.0%), and psoas muscle (0.0%) did not. The VAT volume (3.2 L to 1.6 L) and SAT diameter (3.0 cm to 2.2 cm) also decreased significantly.

Spurny et al. [50] analyzed data from participants of the HELENA trial, a randomized dietary intervention study at the German Cancer Research Centre, Heidelberg, which was undertaken to study the effects of continuous versus intermittent calorie restriction in 150 obese/overweight patients, non-smoking individuals (50% female), between 35 and 65 years without severe, chronic diseases (neither kidney nor liver dysfunction, no major cardiovascular diseases), nor cancer. The study cohort was classified into quartiles based on changes in body weight between the baseline and week 12. For this study, the authors conducted a post hoc analysis to investigate whether moderate weight loss influences BMAT measured by MRI (CSE-WFI). The relative changes in vertebral BMAT (L1–L2) from the baseline to week 12 were 0.0 ± 0.2%, −3.2 ± 0.1%, −6.1 ± 0.2%, and −11.5 ± 0.6% for Q1 (weight loss ≤ 2%) to Q4 (weight loss > 7.5%). Across all four quartiles and for the two-group comparison, Q1 versus Q4, there was a significant difference (*p* < 0.05) for changes in PDFF. Interestingly, despite the found association between weight loss and a decrease in PDFF, there were no associations between PDFF with changes in inflammatory and metabolic biomarkers.

In a more recent study, Ofir et al. [51] investigated BMAT response to lifestyle-induced weight loss in 138 participants of the CENTRAL-MRI trial (mean age 48 years; mean BMI 31 kg/m^2^). The participants were randomized for dietary intervention with or without physical activity. The BMA was quantified by MRI, as well as other fat depots, at the baseline, and at six and eighteen months of intervention. At the baseline, the L3 vertebrae BMAT was positively associated with age, HDL cholesterol, HbA1c, and adiponectin, but not with other fat depots or the other metabolic markers tested. After six months of dietary intervention, the L3 BMAT, but not proximal femur BMAT, declined by an average of 3.1% ± 5.8%, followed by a return to the baseline after eighteen months (*p* < 0.001 and *p* = 0.189 compared to the baseline, respectively). The decrease in BMAT during the first six months was associated with a younger age and a significant reduction in waist circumference, cholesterol, proximal femur BMAT, and SAT. Overall, the BMAT changes did not correlate with changes in other fat depots. Their findings are relevant not only because they combined dietary-induced weight loss with exercise, but also because they demonstrated that BMAT storage and dynamics are largely independent of other fat depots or cardio-metabolic risk markers, highlighting the unique functions of BMAT in humans, which require further exploration.

Unfortunately, bone parameters were not assessed in any of these studies, preventing any conclusions on the relationship between BMAT changes and bone homeostasis in the context of diet-induced weight loss in PwO [48,49,50,51].

Interestingly, in the study performed by Fazeli et al. [25], healthy volunteers underwent a 10-day high-calorie protocol, followed by a 10-day fast. After fasting (mean −8.8% change in weight), they found that the lipid/water ratio at the L4 vertebra, but not at the femoral metaphysis and diaphysis, increased significantly (mean 8.1%, *p* ≤ 0.01), which illustrates the discrepancy between acute and chronic diet-induced weight loss. 

In summary, clinical studies looking at the effect of dietary-induced weight loss on BMAT in the context of PwO are limited and without exploration of bone parameters. Depending on the measurement site (vertebrae and proximal femur), the length of the caloric restriction (4-week to 6-month), and the percentage of the weight loss, BMAT tended to decrease after caloric restriction-induced weight loss. Although weight loss was associated with overall fat loss, a reduction in BMAT did not always follow changes in the fat volumes in other tissues. Although the evidence provided by these studies is limited, it supports the hypothesis that BMAT is not always metabolically or closely linked to other types of fat. However, in contrast to PwO, where BMAT tends to increase, well-controlled target-based dietary-induced weight loss may have a beneficial effect on BMAT that deserves further exploration in larger trials given that high turnover bone loss is a well-known effect of caloric restriction, which means that there is a hypothetic positive correlation between BMD and BMAT in this context.

## 6. Surgical Weight Loss and BMAT

In view of the potential role of BMAT as a regulator of bone metabolism, several research groups have studied the effect of MBS on BMAT, with a follow-up period ranging from 6 to 12 months. The effect of MBS on BMAT may not only represent the effect of weight loss, but may also be influenced by changes in diabetes status and estradiol concentrations, both of which are known to be related to BMAT. Also, the type of MBS, restrictive or malabsorptive, may have differential effects on BMAT, and MBS may have differential effects on specific BMAT sites (vertebrae and tibia). 

### 6.1. Roux-en-Y Gastric Bypass

Kim et al. [52] studied the effect of RYGB surgery after 6 months on vertebral BMAT (L3–L4) in 13 women with and 12 women without diabetes mellitus, with an age of 48 ± 12 years. Approximately one third of the women were postmenopausal. Overall, BMAT did not change, but when stratified according to the presence of diabetes mellitus, BMAT decreased (−6.5 ± 11% absolute change) in women with diabetes and remained stable (+1.8 ± 5.6%) in women without diabetes. HbA1c decreased in both groups, but more extensively in women with diabetes (−1.9 ± 1.1 versus −0.4 ± 0.4%). Trabecular vBMD of the lumbar spine decreased in both groups, but less in women with diabetes (−4.2 ± 6.5%) than in women without (−8.7 ± 4.2%). A negative correlation was found between changes in BMAT and BMD (r = −0.58, *p* < 0.01). In an add-on study consisting largely of the same participants, Kim et al. [53] showed that tibial BMAT did not change, irrespective of diabetes status. In the lumbar spine, the BMAT unsaturated index increased in women with diabetes and decreased in women without diabetes, but in the tibia, there was no difference between the groups (Table 2).

Beekman et al. [54] studied the effect of RYGB after 3 and 12 months on vertebral (L3–L5) BMAT in 14 postmenopausal women without diabetes. Vertebral BMAT was not different 3 months after surgery, but decreased from 50 ± 8% to 46 ± 7% after 12 months compared to the baseline. The lumbar (L3–L4) vBMD decreased from 102 ± 30 mg/cm^3^ at the baseline to 93 ± 23 mg/cm^3^ after 3 months, and 94 ± 28 mg/cm^3^ after 12 months. No correlation between changes in BMAT and changes in vBMD was found, either at 3 or 12 months. 

Blom-Høgestøl et al. [55] studied the effect of RYGB after 12 months on BMAT in 12 men (age 49 ± 11 years) and 18 women (age 45 ± 9 years) of whom 44% were postmenopausal. Moreover, 33% of the men and 39% of the women had type 2 diabetes. In contrast to the other studies reviewed here, BMAT was not determined by MRI, but by histomorphometry of superior iliac crest bone biopsies. The ratio adipocyte volume/marrow volume decreased in women after RYGB (−22% ± 20% relative change), but did not significantly change in men (6.8 ± 38% relative change). The changes in BMAT were not different between subjects with and without diabetes. 

### 6.2. Sleeve Gastrectomy

Bredella et al. [56] studied the effect of SG after 12 months on BMAT in the lumbar spine (L1–L2), femoral diaphysis, and distal tibia metaphysis in adolescents and young adults (age range 13–21 years). Approximately three quarters of the subjects in the SG (*n* = 26) and non-surgical control group (*n* = 26) were female. No data were reported on the pubertal or diabetes status. The lipid-to-water ratio increased in the lumbar spine (0.11 ± 0.13 absolute change) and decreased in the femoral diaphysis (−0.90 ± 2.39 absolute change) and distal tibia metaphysis (−1.33 ± 2.47) after SG, compared to the baseline and controls. The lumbar Tb.vBMD decreased after SG (−6.9 ± 15.5 mg/cm^3^) and did not change in the controls (0.7 ± 13.7 mg/cm^3^). The lumbar saturated BMAT increased after SG, but not in the controls. The lumbar unsaturated BMAT did not change compared to the baseline in both groups, but increased slightly after SG compared to the controls. In the femur and tibia, saturated BMAT decreased after SG compared to the controls, while the change in unsaturated BMAT was not different between the groups. It appears that the increase in lumbar BMAT after SG consists of both saturated and unsaturated BMAT, while the decrease in femoral and tibial BMAT after SG is primarily related to a decrease in saturated BMAT. Huber et al. [57] reported 2-year follow-up data on vertebral BMD and BMAT in 54 adolescents (52 subjects’ baseline data were reported by Bredella et al. [56]), out of which 25 underwent sleeve SG and 29 subjects in a control group (dietary and exercise counselling). This first study to report a follow-up at 2 years, showed an increase in vertebral BMAT and a decrease in vertebral trabecular vBMD, similar to their results after the 1-year follow-up.

### 6.3. Roux-en-Y Gastric Bypass versus Sleeve Gastrectomy

Bredella et al. [58] compared the effect of RYGB (*n* = 11) and SG (*n* = 10) after 12 months on BMAT in the lumbar spine (L1–L2) and femoral diaphysis. The majority of the patients were female (18/21), and the average age was 49 ± 9 and 50 ± 14 years. Two patients in the RYGB group and six patients in the SG had diabetes. No data on the menopausal status or oestradiol concentrations were reported. BMAT in the lumbar spine and femoral diaphysis did not change after RYGB, but increased at both sites after SG. BMAT in the proximal femoral metaphysis did not change irrespective of the bariatric procedure. The lumbar spine vBMD decreased after RYGB and SG, and was not different between groups. 

In a subpopulation of a larger study, Ivaska et al. [59] compared the effect of RYGB (*n* = 7) and SG (*n* = 14) after 6 months on vertebral BMAT (not further specified). The original study population (*n* = 46) consisted largely of women, with a mean age of 45 ± 10 years, 19 with and 27 without diabetes. Age, diabetes, and menopausal status for the subpopulation were not reported. The vertebral BMAT and vBMD did not change after surgery and were not different between RYGB and SG. However, there was a negative correlation between BMAT and vBMD pre- and postoperative, only in subjects without diabetes (r = −0.74, *p* = 0.01 and r = −0.82, *p* = 0.007, respectively). 

Of note, animal models, especially mice, have been proven to be crucial for investigating the mechanisms associated with surgery weight loss [60,61], and their potential translatability to humans has been reviewed by Lutz and Bueter [61]. As in humans, BMAds in mice are not all equals [35,36,62,63]. Indeed, based on the location, cellular properties, and regulation, two distinct subtypes of BMAT, regulated BMAT (rBMAT) and constitutive BMAT (cBMAT), have been distinguished. While rBMAT localizes in the proximal tibia, distal femur, and axial skeleton, and consists of a single or small cluster of BMAds interspersed with hematopoietic cells, cBMAT exists in the distal tibia and caudal vertebrae, and consists of densely packed larger BMAds. Compared to cBMAT that develops rapidly after birth, rBMAT expands progressively throughout life, contains more saturated fatty acids, and expresses low levels of the adipogenic transcription factors Cebpa and Cebpb [62,64]. Consistent with this heterogeneity, the response of BMAT to different stress and altered physiological states is not uniform. Indeed, it is rBMAT (hence the name) that is strictly modulated by different nutritional, environmental, and endocrine factors, and therapeutic interventions. For example, it has been shown to reduce with fasting [65], exercise [66], and surgically induced weight loss [67,68], and to expand in aging [69], and with diabetes [70], and in response to thiazolidinedione treatment [71], HFD [31,32,35,72], and caloric restriction [20,73,74]. However, even though cBMAT is largely resistant to factors known to influence rBMAT, its expansion has been associated to thiazolidinedione treatment [71], and caloric restriction [74,75], and the loss of cBMAds to SG and G-CSF administration [67]. In addition, two recent studies provided a significant contribution to the clarification of the molecular mechanisms associated with bone loss and BM niche remodeling in response to SG. Li et al. [67] reported that SG caused the loss of cortical and trabecular bone, independent of sex, body weight, and diet, the loss of BMAT, and the expansion of myeloid cellularity in association with increased serum levels of G-CSF. The authors also demonstrated that G-CSF was transiently elevated in SG-treated patients and that BMAT loss and myeloid expansion were reduced in G-CSF-null mice. In a further study, Bozadjieva-Kramer et al. [68] showed that intestinal-derived FGF-15 plays a protective role in SG-related bone and BMAT loss. Keeping into mind the caution needed when translating findings from animal models to humans, these studies provide some mechanistic insights, which may be relevant for humans.

In summary, the effects of MBS on BMAT are not consistent among the studies, which is at least partly related to the differences in the study population and duration of the follow-up. Overall, RYGB seems to decrease BMAT in the lumbar spine and iliac crest, in patients with diabetes and postmenopausal women, whereas SG appears to increase lumbar spine BMAT. The effect of MBS on BMAT in lower extremities has been studied less extensively, is highly diverse, and possibly related to the type of surgery and age.

## 7. Conclusions

Bone health impairment in PwO is well known, with normal or elevated BMD, a reduced level of bone remodeling, an increased risk of fracture at specific sites (proximal humerus, ankle, and leg), and changes in certain bone quality parameters [7,38,39,40].

It is interesting to note that, in animal models, the situation is unequivocal, with an expansion of BMAT in HFD conditions, even if the bone modifications of this expansion are variable, as previously stated [30,31,32,33]. In PwO, it is more difficult to draw conclusions on changes in BMAT, mainly because of the paucity and heterogeneity of the data available in the literature. Rather, the trend is for BMAT to increase in subjects with a higher VAT, particularly in pre-menopausal women [40,41,42,43]. No data on postmenopausal women or men over 40 years of age are available. Regarding the link between BMAT and bone parameters, once again considering the limitations mentioned above, the trend is in favor of a negative correlation with increased BMAT levels, lower BMD, and bone quality impairment. However, the local or systemic factors responsible for this negative association remain to be determined.

Following weight loss, either through caloric restriction or MBS, there is no improvement in bone health but, on the contrary, there is deterioration, with a high turnover bone loss, and an increased risk of fracture at traditional osteoporotic sites (wrist and hip), at least in the case of malabsorptive procedures [7,8,9,10,11,12,13,14].

The pathophysiological mechanisms responsible for these bone health alterations in cases of weight loss are only partially understood, and in addition to the usual suspects (mechanical unloading, loss of lean mass, undernutrition, protein insufficiency, vitamin D, and calcium deficiency, etc.) [8,9,10,11,12,13], we need to better understand the involvement of unusual suspects, such as BMAT, given its presumed deleterious role in postmenopausal osteoporosis [15,16,17], diabetes-associated osteoporosis [76,77,78], and bone fragility in patients with chronic kidney disease [79,80,81]. 

In the case of weight loss by caloric restriction, the trend is towards a reduction in BMAT in PwO, subject to sufficient caloric restriction in terms of the duration and induced weight loss [48,49,50,51]. The main limitation of the literature at our disposal is the absence of studies evaluating both variations in BMAT and bone parameters, which makes it impossible to conclude on the imputability of variations in BMAT in the altered bone health observed in this situation, and even less on the factors involved. Moreover, we have no specific data on subjects aged 40 or under in this context.

After surgical weight loss, changes in BMAT are equivocal, depending on age, sex, menopausal status, measurement site, diabetes status, and type of surgery [52,53,54,55,56,57,58,59]. Under these conditions, it is difficult to interpret potential links between changes in BMAT and bone parameters, and even more difficult to discuss the local or systemic factors involved. Lastly, technical issues should be considered when quantifying BMAT and BMD in PwO or in the context of weight loss, as both (changes in) BMAT and (changes in) abdominal fat can influence BMD measurements [82,83,84]. 

Further studies dedicated to assessing changes in BMAT and its association with bone metabolism are needed, either in PwO or in cases of weight loss through caloric restriction or MBS, to better understand the role of BMAT in this context and determine the local or systemic factors involved. To achieve this, it is first and foremost, necessary to determine the population of interest and standardize nomenclature and the methods for the assessment of BMA [29], to make studies comparable between different sites of investigation, or at best to carry out multicenter studies.

We strongly recommend that interventional studies be carried out in high-risk populations, specifically postmenopausal women and men aged ≥50 years. These studies should be conducted over varying time frames, including short term (less than 3 months), medium term (between 3 and 6 months), and long term (more than 6 months), to fully assess the impact on BMAT and bone health over time. It is crucial that these studies employ non-invasive techniques, either WFI or ^1^H-MRS, with multisite evaluations at both the lumbar spine and proximal femur, and multicenter evaluations at least at two different centers. Furthermore, concomitant evaluation of bone parameters should be included in the study design.

## Figures and Tables

**Table 1:** Bone marrow adipose tissue (BMAT) and obesity.

Study	Population	Obesity Classification Study	Imaging Method	Results
Bredella et al., Obesity 2011 [41]	*n* = 47 Age: 32.8 ± 7.1 years BMI (range: 18–41 kg/m^2^) Mean 30 ± 7 kg/m^2^ Female 100% Premenopausal: yes T2D: no	Cross-sectional BMI (range: 18–41 kg/m^2^) Cohort divided into low (*n* = 23) and high visceral fat (VAT) content (*n* = 24)	BMAT: (1H MRS; 3.0T MR imaging system) and QCT for vBMD BMAT in L4 Trabecular vBMD in L4	L4 BMAT as lipid/water ratio Higher in high VAT group vs. low VAT group (*p* = 0.05) Trabecular vBMD (mg/cm^3^) Low VAT group vs. high VAT group (NS)
Bredella et al., Radiology, 2013 [42]	*n* = 106 (46 men and 60 women) Age: 33.7 ± 6.8 years BMI (range 18.1–48.8 kg/m^2^) Mean 33.1 kg/m^2^ ± 7.1 Female: 65% Premenopausal: yes T2D: no	Cross-sectional BMI (range 18.1–48.8 kg/m^2^)	BMAT: (1H MRS; 3.0T MR imaging system) and QCT for vBMD	Positive correlations between BMAT L4 and ectopic fat content independent of BMI Positive correlations between BMAT L4 and HDL cholesterol levels
Cohen et al., JCEM, 2013 [40]	*n* = 40 Age: 37.3 ± 8.2 years BMI: 25.8 ± 4.7 kg/m^2^ Female: 100% Premenopausal: yes T2D: no	Cross-sectional BMI ranged from 20.1 to 39.2 kg/m^2^	BMAT: transiliac bone biopsy, scanned by μCT; evaluation by OsteoMeasure (OsteoMeasure, version 4.00C; OsteoMetrics, Inc., Atlanta, GA, USA) software aBMD (DXA): Femoral neck, total hip, lumbar spine	Increased BMAT in obese and overweight vs. lean controls Negative correlation between BMAT and several bone parameters
Singhal et al., Bone, 2019 [43]	*n* = 60 adolescent girls Age: 14–21 years 18 ± 2 years Female: 100% Premenopausal: yes T2D: no	Cross-sectional BMI above the 95th percentile for age and gender	BMAT: 1H MRS (vertebra): L4 vBMD: QCT L4	BMAT was lower in obese at the femoral diaphysis (*p* ≤ 0.0001) and the lumbar spine (*p* = 0.0039) For the whole group, BMAT at the lumbar spine and femoral diaphysis was inversely associated with BMI, total fat mass, lean mass, and VAT

Abbreviations: T2D: type 2 diabetes mellitus; BMI: body mass index; BMAT: bone marrow adipose tissue; 1H MRS: proton MR spectroscopy; vBMD: volumetric bone mineral density; QCT: quantitative computed tomography; aBMD: areal bone mineral density; DXA: dual-energy X-ray absorptiometry; VAT: visceral adipose tissue.

**Table 2:** Bone marrow adipose tissue (BMAT) and bariatric surgery.

Study	Population	Intervention	Imaging Method	Results
Kim et al. JBMR, 2017 [52]	*n* = 25 Age: 48 ± 12 years Female 100%, Postmenopausal: 37% T2D: 52% Other: Premenopausal women on OAC and postmenopausal women on HRT	RYGB Preoperative and 6 months postoperative	BMAT (1H MRS): L3–4 vBMD (QCT): L3–4	ΔBMAT: Decrease in BMAT in T2D+ group Total: −2.5 ± 10%; *p* = 0.20 T2D+: −6.5 ± 11%; *p* = 0.05 T2D−: +1.8 ± 5.6%; *p* = 0.29 ΔvBMD: Decrease in vBMD of the lumbar spine Total: −6.4 ± 5.9%, *p* < 0.05 T2D+: −4.2 ± 6.5%; *p* < 0.05 T2D−: −8.7 ± 4.2%; *p* < 0.05 Other: In T2D−: negative association between ΔBMAT and Δbody weight Negative correlation between ΔBMAT and ΔBMD (r = −0.58, *p* < 0.01) Greater decrease in HbA1c was associated with decrease in BMAT
Kim et al. Bone Reports 2022 [53]	*n* = 25 Age: 48.2 ± 11.7 years Female 100%, Postmenopausal: 37% T2D: 52% Other: Premenopausal women on OAC and postmenopausal women on HRT	RYGB Preoperative and 6 months postoperative	BMAT: (1H MRS) BMAT and unsaturated lipid index (UI) L3–4 and BMAT in the distal tibia in subset of *n* = 15 vBMD: QCT L3–4	Spine ΔBMAT: No change Total: 66 ± 14% → 65 ± 14; −1.5%, *p* = 0.54 T2D+: 66 ± 13% → 62 ± 14; −6.5%, *p* = 0.09 T2D−: 65 ± 15% → 68 ± 14; +4%, *p* = 0.13 Spine ΔUI: Increased in T2D+ Total: 5.1 ± 1.7% → 5.1 ± 1.6%; 0%, *p* = 0.90 T2D+: 4.5 ± 0.8% → 5.6 ± 1.5%; +24.4%, *p* = 0.02 T2D−: 5.7 ± 2.2% → 4.6 ± 1.5%; −19.3%, *p* = 0.06 Tibia (*n* = 15; T2D+ *n* = 9) ΔBMAT: No change Total: 98 ± 1% → 98 ± 1%; −0.3%, *p* = 0.32 T2D+: 97 ± 1% → 97 ± 1%; −0.4%, *p* = 0.34 T2D−: 98 ± 1% → 98 ± 1%; −0.1%, *p* = 0.86 Tibia (*n* = 15; T2D+ *n* = 9) ΔUI: Decreased in total group Total: 4.2 ± 1.2% → 3.9 ± 1.2%; −7.1%, *p* = 0.04 T2D+: 4.1 ± 1.0% → 3.8 ± 1.2%; −7.3%, *p* = 0.24 T2D−: 4.5 ± 1.5% → 3.9 ± 1.3%; −13.3%, *p* = 0.11 vBMD L3–L4: Decreased in all groups Total: −6.4 ± 5.9%, *p* < 0.05 T2D+: −4.2 ± 6.5%, *p* < 0.05 T2D−: 8.7 ± 4.2%, *p* < 0.05 Other: At the spine, a significant interaction between T2D status and ΔBMAT (*p* = 0.02) and ΔUI (*p* < 0.01) ΔUI was inversely correlated with ΔHbA1c (r = −0.47, *p* = 0.02)
Beekman et al. Obesity, 2021 [54]	*n* = 14 Age: 58 ± 4 years Female: 100% Postmenopausal: 100% T2D: 0%	RYGB Preoperative, postoperative (3 and 12 months)	BMAT (CSE-WFI) L3–5 vBMD (QCT) L3–4	ΔBMAT: Decreased 12 months after surgery Baseline: 51 ± 8% 3 months: 50 ± 8% 12 months: 46 ± 7%; −9.3%, *p* = 0.004 ΔvBMD: Decreased 3 and 12 months after surgery Baseline: 101 ± 26 mg/cm^3^ 3 months: 94 ± 28 mg/cm^3^; −7.6%, *p* = 0.003 12 months: 94 ± 28 mg/cm^3^; −6.4%, *p* = 0.035 Other: No correlation between changes in BMAT and changes in vBMD Positive correlation between changes in BMAT and changes in body weight
Blom-Høgestøl et al. JBMR 2019 [55]	*n* = 30 Age: 46.3 ± 9.6 years Female: 60% Postmenopausal: *n* = 8; 44% of the females T2D: 37%	RYGB Preoperative and 1-year postoperative	BMAT: Iliac crest biopsy (grid-based point counting) aBMD: lumbar spine and femoral neck	ΔBMAT: BMAT decreased after RYGB in female subjects Total: 40.4 ± 1.7% → 35.6 ± 12.8%; −10,7%, *p* = 0.04 Female: 39.4 ± 9.9% → 30.1 ± 9.0%; −22.4% *p* < 0.001 Male: 41.9 ± 8.4% → 43.7 ± 13.8%; +6.8%, ns ΔBMAT: BMAT decreased similar in pre- and postmenopausal women Postmenopausal: −18.8 ± 18% Pre-menopausal: −25.3 ± 21.3% ΔBMAT: BMAT decreased in T2D+ and T2D− groups T2D+: 43.3 ± 10.9% → 40.3 ± 15.3%; −6.9% T2D−: 38.7 ± 8.1% → 32.8 ± 10.7%; −15.2% (*n* = 11, after RYGB: remission in 10/11) ΔaBMD: decreased after RYGB Lumbar spine: −4.3 ± 5.9% Femoral neck: −8.2 ± 4.85% Total hip: −11.8 ± 4.9% Other: Changes in serum estradiol in males was negatively associated with changes in BMAT after RYGB (Serum testosterone increased, *p* < 0.001, estradiol decreased, *p* = 0.035) ΔBMAT was positively associated with ΔBMI and Δbody fat
Bredella et al. JCEM, 2020 [56]	*n* = 26 Age: 18.0 ± 2.1 years Female: 73% Postmenopausal: 0% T2D: unknown	Sleeve gastrectomy (SG) Preoperative and 1-year postoperative	BMAT: (1H MRS) BMAT and unsaturated and saturated BMAT (lipid to water ratio): L1–L2, femoral mid-diaphysis, distal tibia metaphysis vBMD (QCT): L1–L2	Lumbar spine L1–L2: Decrease in BMAT and increase in saturated lipids ΔBMAT: 0.37 ± 0.17 → 0.49 ± 0.25; +32%, *p* = 0.001 ΔUnsaturated 0.03 ± 0.01 → 0.04 ± 0.03; +33%, *p* = 0.1 ΔSaturated 0.30 ± 0.15 → 0.39 ± 0.19; +30%, *p* < 0.001 Femoral mid-diaphysis: BMAT unchanged and decrease in unsaturated lipids ΔBMAT: 4.89 ± 2.96 → 3.92 ± 2.33; −20%, *p* = 0.09 ΔUnsaturated 0.43 ± 0.29 → 0.30 ± 0.24; −30%, *p* = 0.02 ΔSaturated 3.35 ± 1.95 → 2.93 ± 1.73; −13%, *p* = 0.3 Tibia distal (metaphysis): Decrease in BMAT and decrease in saturated lipids ΔBMAT: 11.39 ± 3.02 → 10.01 ± 2.00; −12%, *p* = 0.04 ΔUnsaturated 1.31 ± 0.62 → 1.45 ± 0.76; +11%, *p* = 0.39 ΔSaturated 8.82 ± 2.41 → 7.72 ± 1.65; −12%, *p* = 0.02 vBMD L1–L2: Decrease in vBMD 200 ± 39 mg/cm^3^ → 193 ± 38 mg/cm^3^; −3%, *p* = 0.04
Huber et al. Radiology, 2023 [57]	*n* = 54 Age 18 ± 3 years, range 13–24 years Female: 41 T2D: unknown Postmenopausal: none	SG: *n* = 25 Control: *n* = 29 (dietary and exercise counselling) Preoperative and after 24 months	BMAT L1–L2 vBMD L1–L2	Increase in BMAT 2 years after SG ΔBMAT: SG 0.38 ± 0.15 → 0.48 ± 0.19; +26% *p* = 0.001 Controls 0.46 ± 0.22 → 0.52 ± 0.25; +13% *p* = 0.11 *p*-value for 24 months change between groups: *p* = 0.40 Decrease in vBMD after SG ΔvBMD (trabecular) mg/cm^3^: SG 250 ± 28 → 232 ± 29; −7.2% *p* < 0.001 Controls 242 ± 34 → 241 ± 33; −0.4% *p* = 0.99 *p*-value for 24 months change between groups: *p* < 0.001 Inverse correlation between ΔBMAT and ΔvBMD (r = −0.41 *p* = 0.01).
Bredella, Bone, 2017 [58]	*n* = 21 Age 49 ± 9 years Female: 86% Postmenopausal: Unknown T2D: 38%	RYGB: *n* = 11 T2D: *n* = 2 (18%) SG: *n* = 10 T2D: *n* = 6 (60%) Preoperative and 1-year postoperative	BMAT: (1H MRS) L1–L2 (LS); femur (metaphysis and diaphysis) vBMD: QCT L3–L4, femoral neck and total hip	ΔBMAT (data estimated from graph): Increase in BMAT of the lumbar spine and femoral diaphysis after SG RYGB: Lumbar spine: −6%; ns Diaphysis: −2%; ns Metaphysis: +2%; ns SG: Lumbar spine +20%; *p* < 0.05 Diaphysis: +18%; *p* < 0.05 Metaphysis: −6%; ns vBMD: Decrease after both RYGB and SG Other: No difference between T2D+ and T2D− Excluding male subjects, did not change the results Positive correlation between ΔBMI/VAT and ΔBMAT
Ivaska et al. Bone, 2017 [59]	*n* = 18 (subpopulation) Age: unknown (whole group: 45 ± 9.5 years) Female: unknown (whole group: *n* = 42, 91%) T2D: *n* = 8 (38%) Postmenopausal: unknown	RYGB: *n* = 7 SG: *n* = 14 Preoperative and 6 months postoperative	BMAT: 1H MRS (vertebra) vBMD: QCT L1–L2, VAT	ΔBMAT: No difference in BMAT after RYGB or SG Total: median change −10.7%, *p* = 0.18 Negative correlation between BMAT and vBMD pre- and postoperative, in subjects T2D− (r = −0.74, *p* = 0.01 and r = −0.82, *p* = 0.007, respectively) No correlation between BMAT and vBMD in T2D+ Exact number of subjects with BMAT measurements remains unclear, data only in graph, no exact data on ΔBMAT in table or graph Unclear in which vertebral body BMAT was quantified

Abbreviations: T2D: type 2 diabetes mellitus; OAC: oral contraceptives; HRT: hormone replacement therapy; RYGB: Roux-en-Y gastric bypass; BMAT: bone marrow adipose tissue; 1H MRS: proton MR spectroscopy; UI: unsaturated lipid index; CSE-WFI: chemical shift encoding-based water–fat MRI; vBMD: volumetric bone mineral density; QCT: quantitative computed tomography; SG: sleeve gastrectomy; aBMD: areal bone mineral density; VAT: visceral adipose tissue.
